# Targeting Endogenous Antioxidants to Prevent Cardiovascular Diseases

**DOI:** 10.1161/JAHA.112.005215

**Published:** 2012-12-19

**Authors:** W. H. Wilson Tang

**Affiliations:** Center for Cardiovascular Diagnostics and Prevention, Department of Cellular and Molecular Medicine, Lerner Research Institute, Cleveland ClinicCleveland, OH; Department of Cardiovascular Medicine, Heart and Vascular Institute, Cleveland Clinic, Cleveland, OH

**Keywords:** editorials, cardiovascular diseases, oxidative stress plasma, peroxiredoxin, prognosis, risk stratification

Oxidative stress has long been associated with a wide range of cardiovascular risk factors and likely contributes to the progression of cardiovascular diseases in animal models and in humans.^1^ Numerous lines of evidence indicate that pathways of oxidative and nitrative stress can contribute to cardiovascular disease via multiple mechanisms, such as through formation of atherogenic lipoproteins, initiation of lipid oxidation, and propagation of damage to endothelial cells and myocytes.^1^ Nevertheless, quantifying the degree of oxidative stress has posed major challenges because many of these processes occur intracellularly and involve complicated issues with stability and variability in biospecimens. Hence, the identification of biomarkers of oxidative stress has focused on detecting systemic stable oxidized products (eg, oxidized low-density lipoprotein, F2-isoprostanes) or identifying the presence of mediators of oxidative stress pathways (eg, oxidation of nitric oxide by myeloperoxidase^2^ and ceruloplasmin^3^). In addition, quantifying the activities of endogenous antioxidant proteins such as high-density lipoprotein–associated paraoxonase-1 activities^4^ or glutathione^5^ may reflect underlying oxidative imbalance. Indeed, large prospective cohorts have identified individuals with abnormal levels of several of the aforementioned biomarkers to be at greater risk for future cardiovascular risks.^2–5^

Recently, a family of proteins called “peroxiredoxins” was described in various organisms.^6^ Peroxiredoxins are ubiquitously synthesized to reduce hydrogen peroxide and alkyl hydroperoxides to water and alcohol with the use of reducing equivalents derived from thiol-containing donor molecules.^7^ Peroxiredoxins has also been postulated to play a regulatory role in the activation of the transcription factor nuclear factor-κB,^8^ and prevent the production of reactive oxygen species induced by epidermal growth factor or p53.^9^ Although the majority of peroxiredoxin proteins are intracellularly localized, peroxiredoxin-IV (Prx4, encoded by the gene) is the only known secretory form located in the extracellular space.^10^ Hence, the ability to detect circulating levels of Prx4 holds promise in quantifying oxidative stress for risk stratification. Evidence for peroxiredoxins in the development of cardiovascular diseases is emerging.^11,12^ In particular, the transgenic mouse model of human *PRDX4* demonstrated protection of pancreatic beta cells against streptozotocin-induced injury^13^ as well as prevention of atherosclerosis formation in apolipoprotein E–null mice^14^ via suppression of oxidative stress and inflammatory signaling. These findings have provided promise that circulating Prx4 may have direct capabilities in cardiovascular protection, particularly in inflammatory disease states.^15^

In the October issue of *JAHA*, Abbasi and colleagues measured serum levels of Prx4 in the large Prevention of Renal and Vascular End-stage Disease study that included middle-aged subjects with microalbuminuria. They observed increased levels of Prx4 being associated with risk factors for cardiovascular diseases as well as incident development of cardiovascular events, even though the overall range of Prx4 levels was relatively low in this primary prevention cohort.^16^ It appears that Prx4 has a complex relationship with incident cardiovascular risk, whereby both very low and very high levels portend poorer outcomes. This is in direct contrast with other known endogenous antioxidants such as paraoxonase-1, whereby detection of low activity levels portends poorer outcomes.^4^ The authors hypothesized that low levels demonstrated insufficient antioxidant responses and high levels are suggestive of compensatory response toward heightened oxidative stress. In other words, Prx4 may better reflect underlying antioxidant response rather than underlying oxidative vulnerability; hence, these findings may hint that it is less likely for Prx4 (or the lack of) to function as a direct mediator of oxidative stress.

How can we translate these new findings to the bedside? Although overall there appears to be incremental prognostic value with Prx4 levels in predicting incident cardiovascular events, the authors did concede that *PRDX4* levels provided only marginal reclassification of risk above and beyond Framingham risk score. This was also apparent when the prognostic value was significantly attenuated by other systemic markers of inflammation like high-sensitivity C-reactive protein. Hence, it is still unclear how quantification of Prx4 can add to the current clinical practices for purposes of identifying individuals at increased cardiovascular risk, particularly when there is established evidence to support therapeutic intervention targeting other inflammatory markers, like high-sensitivity C-reactive protein.^17,18^ Regardless of clinical utility, these findings have provided validation for a broader concept that heightened oxidative stress is contributory to cardiovascular disease progression and that circulating Prx4 may play a cardiovascular protective role.^19^

The contribution of oxidative stress in human disease states has long been recognized, yet the concept of “antioxidant” is often used in a vague manner without much appreciation of its complexity. Bolstering antioxidative pathways not only may benefit cardiovascular health but also may prevent many other chronic diseases such as cancer and diabetes mellitus; hence, understanding how to enhance them holds great promise. Showing the association between a biomarker with a postulated mechanism with severity of a disease state is only the first step. The intriguing association of Prx4 levels and cardiovascular risk as demonstrated by Abbasi et al has certainly established the relevance of this antioxidative pathway in humans. There are many questions that remain unanswered. How it relates and/or compares to other biomarkers of inflammation or oxidative stress remains to be determined. What directly or indirectly affects its synthesis, release, and breakdown in humans is still not well understood in humans. What pharmacologic or nonpharmacologic treatment interventions can modulate circulating Prx4 levels are also unclear at this point. Taken together, the ability to detect circulating Prx4 levels will likely open to a number of exciting new mechanistic insights into the role of peroxiredoxins in human cardiovascular diseases. It is therefore imperative for future investigations to determine if biomarkers of oxidative stress (Prx4 and others) can track favorable effects with effective interventions. It is important to demonstrate that targeting modifiable risk factors and encouraging lifestyle modifications such as dietary changes and exercise based on abnormal biomarker level can lead to interval improvement of levels of oxidative stress and subsequent risk reduction. Hence, future investigations in the determinants of Prx4 expression are warranted with the hope to identify therapeutic interventions that may enhance endogenous antioxidant activities for cardiovascular protection.
